# Butyrate Differentiates Permissiveness to Clostridioides difficile Infection and Influences Growth of Diverse C. difficile Isolates

**DOI:** 10.1128/iai.00570-22

**Published:** 2023-01-24

**Authors:** Daniel A. Pensinger, Andrea T. Fisher, Horia A. Dobrila, William Van Treuren, Jackson O. Gardner, Steven K. Higginbottom, Matthew M. Carter, Benjamin Schumann, Carolyn R. Bertozzi, Victoria Anikst, Cody Martin, Elizabeth V. Robilotti, Jo May Chow, Rachael H. Buck, Lucy S. Tompkins, Justin L. Sonnenburg, Andrew J. Hryckowian

**Affiliations:** a Department of Medicine, Division of Gastroenterology and Hepatology, University of Wisconsin School of Medicine and Public Health, Madison, Wisconsin, USA; b Department of Medical Microbiology & Immunology, University of Wisconsin School of Medicine and Public Health, Madison, Wisconsin, USA; c Department of Microbiology & Immunology, Stanford University School of Medicine, Stanford, California, USA; d Microbiology Doctoral Training Program, University of Wisconsin—Madison, Madison, Wisconsin, USA; e Department of Chemistry, Stanford University, Stanford, California, USA; f Howard Hughes Medical Institute, Stanford University, Stanford, California, USA; g Department of Pathology, Stanford University School of Medicine, Stanford, California, USA; h Department of Bacteriology, University of Wisconsin—Madison, Madison, Wisconsin, USA; i Department of Medicine, Division of Infectious Disease, Stanford University School of Medicine, Stanford, California, USA; j Abbott, Nutrition Division, Columbus, Ohio, USA; k Chan Zuckerberg BioHub, San Francisco, California, USA; University of Illinois at Chicago

**Keywords:** bacterial pathogenesis, microbial metabolites, microbiome, microbiota

## Abstract

A disrupted “dysbiotic” gut microbiome engenders susceptibility to the diarrheal pathogen Clostridioides difficile by impacting the metabolic milieu of the gut. Diet, in particular the microbiota-accessible carbohydrates (MACs) found in dietary fiber, is one of the most powerful ways to affect the composition and metabolic output of the gut microbiome. As such, diet is a powerful tool for understanding the biology of C. difficile and for developing alternative approaches for coping with this pathogen. One prominent class of metabolites produced by the gut microbiome is short-chain fatty acids (SCFAs), the major metabolic end products of MAC metabolism. SCFAs are known to decrease the fitness of C. difficile *in vitro*, and high intestinal SCFA concentrations are associated with reduced fitness of C. difficile in animal models of C. difficile infection (CDI). Here, we use controlled dietary conditions (8 diets that differ only by MAC composition) to show that C. difficile fitness is most consistently impacted by butyrate, rather than the other two prominent SCFAs (acetate and propionate), during murine model CDI. We similarly show that butyrate concentrations are lower in fecal samples from humans with CDI than in those from healthy controls. Finally, we demonstrate that butyrate impacts growth in diverse C. difficile isolates. These findings provide a foundation for future work which will dissect how butyrate directly impacts C. difficile fitness and will lead to the development of diverse approaches distinct from antibiotics or fecal transplant, such as dietary interventions, for mitigating CDI in at-risk human populations.

**IMPORTANCE** Clostridioides difficile is a leading cause of infectious diarrhea in humans, and it imposes a tremendous burden on the health care system. Current treatments for C. difficile infection (CDI) include antibiotics and fecal microbiota transplant, which contribute to recurrent CDIs and face major regulatory hurdles, respectively. Therefore, there is an ongoing need to develop new ways to cope with CDI. Notably, a disrupted “dysbiotic” gut microbiota is the primary risk factor for CDI, but we incompletely understand how a healthy microbiota resists CDI. Here, we show that a specific molecule produced by the gut microbiota, butyrate, is negatively associated with C. difficile burdens in humans and in a mouse model of CDI and that butyrate impedes the growth of diverse C. difficile strains in pure culture. These findings help to build a foundation for designing alternative, possibly diet-based, strategies for mitigating CDI in humans.

## INTRODUCTION

Clostridioides difficile is an opportunistic diarrheal pathogen and is an “urgent threat” to global health, as it causes over 220,000 cases and 13,000 deaths per year in the United States alone (1). A disrupted (dysbiotic) gut microbiome, most commonly resulting from antibiotic use, is the primary risk factor for C. difficile infection (CDI) (2), highlighting the gut microbiome as a key mediator of CDI. Therefore, measures to positively impact the composition and function of the gut microbiome represent potential approaches to understand and mitigate C. difficile pathogenesis.

Diet is one of the most powerful ways to impact the composition and function of the gut microbiome (3, 4). A growing body of literature demonstrates that dietary changes impact C. difficile, the microbiome, and the host during animal models of CDI. For example, low-protein diets are protective against CDI and high-fat/high-protein diets exacerbate CDI (5, 6), and the availability of the amino acid proline in particular impacts C. difficile fitness in murine models (7). Dietary fat promotes C. difficile-mediated mortality in mice (8). Diets containing inulin, xanthan gum, and complex mixtures of microbiota-accessible carbohydrates (MACs) reduce C. difficile burdens below detection in mice (9, 10), and fructooligosaccharides (FOS) increase survival in infected hamsters (11). Another carbohydrate, trehalose, increases CDI mortality in mice (12) but does not impact C. difficile burdens or virulence in chemostats containing human-derived microbiomes (13). Finally, the abundance of metals such as zinc also correlates with several measures of CDI severity in mice (14). Together, these studies are part of a growing literature that supports that microbiome- and host-dependent metabolite availability in the gut, rather than a specific “susceptible” or “resistant” microbiome configuration, defines colonization resistance to C. difficile (9, 15–18). Furthermore, each of the abovementioned diet-driven impacts on CDI represents an opportunity to understand the diverse metabolic requirements of, and niches occupied by, C. difficile during CDI and is likely to lead to the development of new concepts and approaches for mitigating CDI in at-risk human populations. Notably, this previous work was carried out under controlled experimental conditions which were designed to specifically manipulate conditions of interest using animal models of CDI and a limited number of C. difficile strains. Therefore, though animal models of CDI recapitulate many relevant aspects of human disease, it is unclear to what extent these findings translate to human populations who are infected by phylogenetically diverse C. difficile strains and who differ in important parameters like immune status and dietary habits.

Of the dietary inputs described above which impact CDI, MACs represent a particularly high-yield avenue for diet-focused work on C. difficile. In particular, the short-chain fatty acids (SCFAs), which are the metabolic end products of MAC metabolism by the microbiome (19), impact C. difficile fitness in pure culture and in animal models of infection (9, 20, 21) and have pleiotropic beneficial effects on the host (22–28). Three SCFAs (acetate, propionate, and butyrate) are the most abundant metabolites in the gut, together reaching concentrations of over 100 mM in the gastrointestinal tracts of humans (29), and are influenced by host MAC consumption. The dysbiotic conditions which favor CDI are characterized by low SCFA concentrations in both humans and animal models (9, 12, 16, 30, 31).

Despite the emerging understanding of the impact of dietary MACs and their metabolic end products on CDI and the promise for rapid translation to humans, key questions remain. For example, which MACs are most effective in ameliorating CDI? What parameters differentiate effective MACs from ineffective MACs? What mechanism(s) underlies these differences? Are these conclusions generalizable to all C. difficile strains? To begin to answer these questions, this study leverages a murine model of CDI, human samples, and a collection of C. difficile isolates to demonstrate that elevated concentrations of butyrate are associated with a reduction in C. difficile fitness in pure culture, in mice, and in humans. Together, these findings provide the foundation for future work aimed at understanding the metabolic interactions that dictate C. difficile fitness and pathogenesis and for developing new approaches to mitigate CDI in at-risk human populations.

## RESULTS

### Inulin and FOS differentially impact C. difficile burdens in mice.

**FIG 1:**
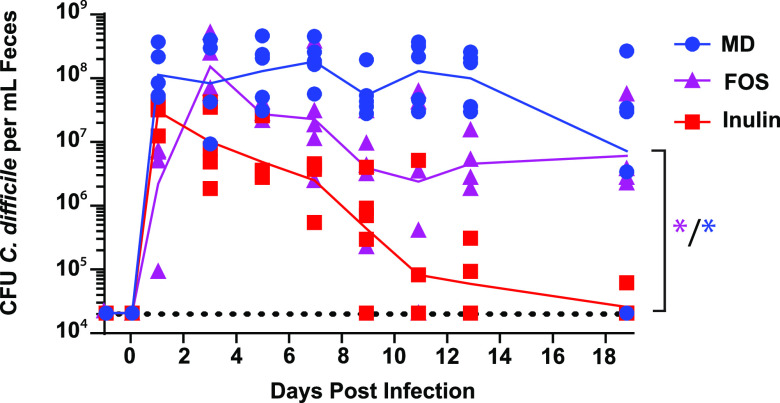
A diet containing inulin, but not FOS, as the sole MAC source reduces C. difficile 630 colonization below detection. Mice were fed a MAC-deficient (MD) diet or diets containing inulin or FOS as the sole MAC source and were subjected to murine model CDI. Burdens of C. difficile in mouse feces were quantified until 19 days postinfection and are shown as blue circles for mice fed the MD diet, purple triangles for mice fed the FOS-containing diet, and red squares for mice fed the inulin-containing diet. The geometric means of C. difficile burdens for mice fed each diet are connected by lines matching this diet-specific coloring scheme. The limit of detection of the C. difficile quantification assay (20,000 CFU C. difficile/mL feces) is shown as a horizontal dotted black line. C. difficile burdens in feces are significantly different between mice fed the inulin-containing diet and mice fed the MD diet or the FOS-containing diet (assessed at the experimental endpoint via Mann-Whitney test; *, *P* < 0.05).

In previous work, we demonstrated that inulin, a β-2,1-linked fructan, suppresses C. difficile burdens in a murine model of CDI (9). This model utilizes clindamycin as a clinically relevant antibiotic perturbation and, in the context of mice fed a MAC deficient diet, replicates a state of persistent colonization with moderate inflammation commonly observed in the clinic (32). To begin to test the generalizability of the findings with inulin to other purified MAC sources, we focused on FOS, which is structurally identical to inulin except for its degree of polymerization (DP) (FOS DP = 2 to 8 and inulin DP = 2 to 60) (33). In contrast to mice fed inulin, mice fed FOS retain high burdens of C. difficile 630 during CDI with no significant difference from the MAC-deficient (MD) diet at day 19 (Fig. 1). These results lead to two possible hypotheses, that the effect of MAC sources on C. difficile burden is driven either by MAC effects on the microbiota or by direct effects on C. difficile.

**FIG 2:**
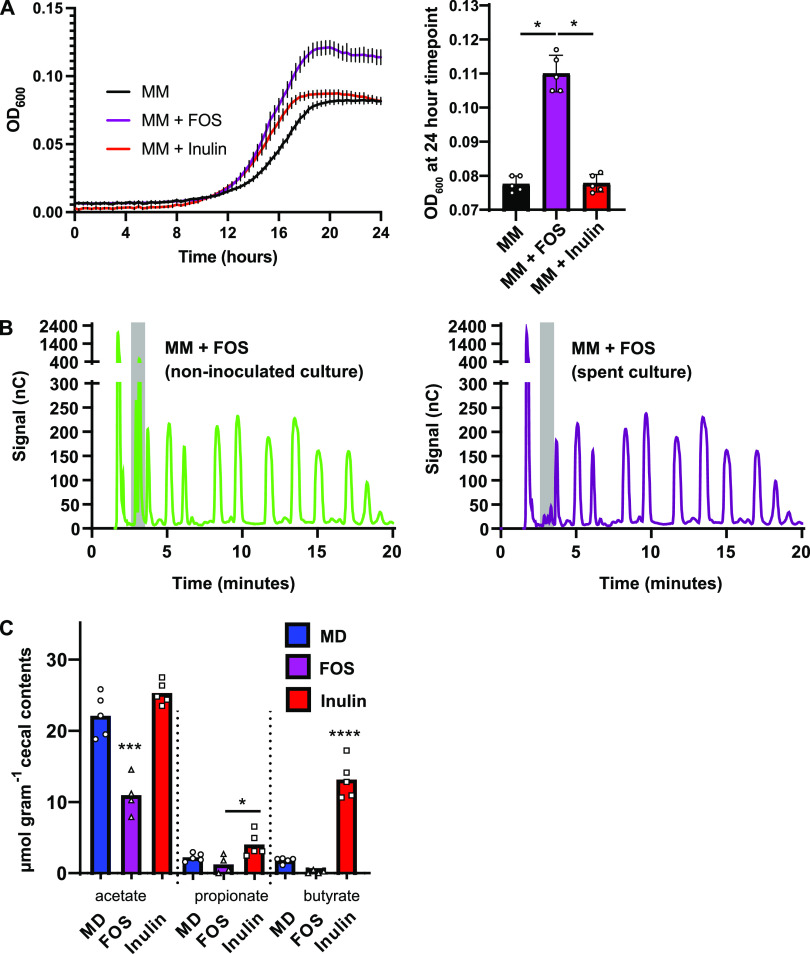
Growth of C. difficile 630 on FOS *in vitro* and differential impacts of FOS and inulin on SCFA production by the microbiome in C. difficile 630-infected mice. (A) C. difficile 630 was grown in PETC-F minimal medium (MM, black lines), MM + FOS (purple lines), and MM + inulin (red lines) (*n* = 5 biological replicates per strain), for 24 h, and culture density (OD_600_) was monitored (left). Lines and error bars represent mean OD_600_ readings and standard deviation at each time point, respectively. Statistically significant differences between the final OD_600_s of these cultures were determined by Mann-Whitney test (*, *P* < 0.05). (B) Filtered supernatants from these cultures were analyzed using high-performance anion-exchange chromatography and a pulsed amphoteric detector (HPAEC-PAD). Representative chromatograms are shown for uninoculated media (left, green) and for spent media (right, purple). See Fig. S1 in the supplemental material for a reference chromatogram, demonstrating that the metabolites depleted by C. difficile are monosaccharides that contaminate the FOS preparation rather than FOS itself. (C) The SCFAs acetate, propionate, and butyrate were quantified in the cecal contents of mice described in the legend to Fig. 1, collected after euthanasia at 19 days postinfection. Individual data points represent SCFA concentrations measured via GC-MS, and bars represent mean concentrations. Blue bars represent SCFAs quantified in mice fed the MAC-deficient diet, purple bars represent SCFAs quantified in mice fed the FOS-containing diet, and red bars represent SCFAs quantified in mice fed the inulin-containing diet. Statistical significance was determined by one-way analysis of variance with Tukey’s multiple-comparison test (*, *P* < 0.05; ***, *P* < 0.001; ****, *P* < 0.0001). See also Fig. S1.

To begin to understand the differential impacts of these two MAC types on CDI, we grew C. difficile 630 in minimal medium supplemented with FOS or inulin. C. difficile 630 grows to a higher density in minimal medium supplemented with FOS than in minimal medium supplemented with inulin (Fig. 2A). This and previous work demonstrate that C. difficile cannot use inulin for growth (34). However, these data suggest that C. difficile may be able to utilize FOS via an uncharacterized glycoside hydrolase (GH). Of the glycoside hydrolase classes which can metabolize fructans, the only class present in C. difficile 630 is a GH32 family member, encoded by CD630_18050 (35). GH32 enzymes are important for fructan hydrolysis and are highly specific for their substrates (e.g., inulin, FOS, levan, and sucrose) (36). To address the hypothesis that FOS does not suppress CDI because C. difficile metabolizes FOS via a FOS-specific GH32 enzyme, we performed high-performance anion-exchange chromatography with pulsed amperometric detection (HPAEC-PAD) to determine the extent of FOS utilization by C. difficile grown in FOS-supplemented minimal medium. We determined that C. difficile does not utilize FOS but instead consumes the trace amounts of glucose and fructose in the FOS preparation (Fig. 2B; peaks within gray bars correspond to glucose and fructose based on reference chromatograms in Fig. S1 in the supplemental material). Therefore, this work supports previous findings that C. difficile does not readily consume MACs (34) and that it is likely that factors unrelated to FOS metabolism by C. difficile contribute to the inability of FOS to clear murine CDI.

The major metabolic end products of MAC metabolism by the gut microbiome are SCFAs, predominantly acetate, propionate, and butyrate (19, 37, 38). Based on the metabolic capabilities of a given microbiome, MACs can differentially impact SCFA abundance and ratios in the gut. Our previous work and the work of others showed that SCFAs influence the fitness of C. difficile in animal models and in culture (9, 21, 30, 39) and that FOS and inulin differentially impact the quantities and proportions of SCFAs produced by gut microbes *in vitro* (40). We therefore hypothesized that FOS and inulin differentially impact CDI based on the quantities and types of SCFAs produced by the microbiome during infection. To address this hypothesis, we quantified acetate, propionate, and butyrate in the cecal contents of conventional mice fed FOS, inulin, or a MAC-deficient diet (Fig. 1) as described previously (9). Mice fed FOS have lower levels of acetate, propionate, and butyrate in their ceca than do those fed inulin (Fig. 2C). In addition, less acetate was detected in the cecal contents of FOS-fed mice than in the contents of mice fed a MAC-deficient diet (Fig. 2C), suggesting that alternative metabolic end products, distinct from acetate, propionate, and butyrate, are produced by FOS-fed microbiomes in this model. Consistent with our previous work, these data suggest that MACs that favor an SCFA-enriched gut environment discourage CDI.

### Cecal butyrate concentrations differentiate mice that do and do not suppress CDI across diverse MAC types.

**FIG 3:**
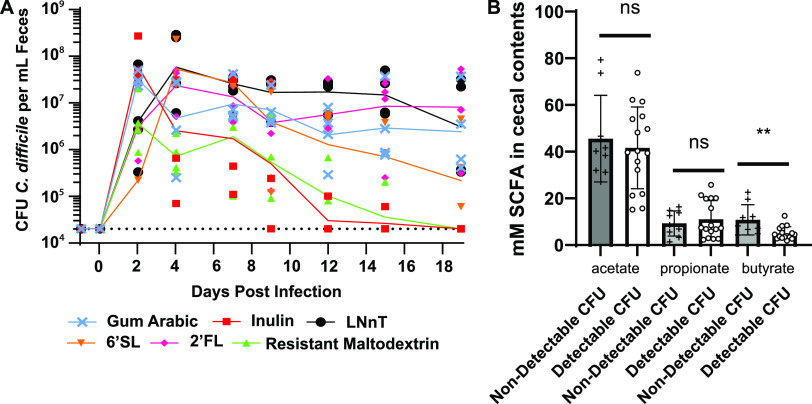
Differential impacts of MACs on C. difficile burdens and an association of C. difficile 630 clearance with cecal butyrate concentrations in mice. (A) Burdens of C. difficile in mouse feces were quantified until 19 days postinfection and are shown as light blue crosses for mice fed the gum arabic diet, red squares for mice fed the inulin-containing diet, light green upward triangles for mice fed the resistant maltodextrin diet, orange downward triangles for mice fed the 6′-SL diet, magenta diamonds for mice fed the 2′-FL diet, and black circles for mice fed the LNnT diet. The geometric means of C. difficile burdens for mice fed each diet are connected by lines matching this diet-specific coloring scheme. The limit of detection is displayed as a dotted line. (B) The SCFAs acetate, propionate, and butyrate were quantified in cecal contents collected from mice after euthanasia at 19 days postinfection via LC-MS. Individual measurements are shown as circles, squares, and triangles (acetate, propionate, and butyrate, respectively) and are stratified by mice that had detectable C. difficile in their feces versus those that had undetectable C. difficile in their feces. Means are displayed as bars, standard deviations are displayed as error bars, and statistical significance was assessed by Mann-Whitney test (**, *P* < 0.01; ns, not significant).

The conclusions that elevated SCFAs negatively impact C. difficile burdens in the mouse gut are based on experiments that used a limited number of dietary conditions. Specifically, both a complex MAC-rich diet (5010 Purina LabDiet) and a diet containing inulin as the sole MAC source suppress CDI. On the other hand, MAC-deficient diets or a diet containing FOS as the sole MAC source do not clear CDI (Fig. 1) (9). To further generalize these findings, we fed 5 additional diets containing different MAC sources to mice with experimental CDI. These diets contained one of three individual human milk oligosaccharides (HMOs; 2′-fucosyllactose [2′-FL], 6′-siaylyllactose [6′-SL], and lacto-*N*-neotetraose [LNnT]), a digestion-resistant maltodextrin, or a complex mixture of MACs found within gum arabic. These MACs were selected (i) based on evidence that HMOs impact SCFA production by gut microbes (41) and have a variety of beneficial effects on the eukaryotic host (42) and (ii) to understand whether the SCFAs produced by other structurally unrelated plant polysaccharides (distinct from fructans or the complex mixture of MACs present in standard rodent diets) impact C. difficile infection. We observed that these MAC types differentially impact C. difficile burdens and that out of these additional MACs tested, maltodextrin was the only one that consistently reduced C. difficile burdens below detection (Fig. 3A). We then quantified acetate, propionate, and butyrate in the cecal contents of mice shown in Fig. 3A to determine if SCFA concentrations differentiate mice with and without detectable fecal C. difficile in this cohort of mice fed inulin, gum arabic, resistant maltodextrin, 6′-SL, 2′-FL, and LNnT. The mice with detectable C. difficile in their stool at the experimental endpoint had significantly lower butyrate concentrations in their cecal contents than mice with no detectable C. difficile in their stool (mean butyrate concentrations of 3.9 mM and 10.8 mM, respectively) (Fig. 3B). This diet-agnostic analysis of SCFA levels demonstrates that mice that cleared C. difficile below detection have significantly elevated levels of butyrate (but not acetate or propionate) in their cecal contents relative to mice with detectable C. difficile.

### Fecal butyrate concentrations differentiate stool samples from humans with and without CDI.

**FIG 4:**
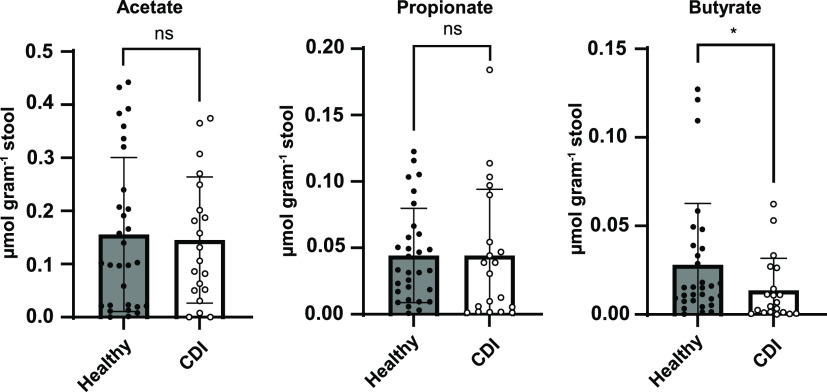
Fecal butyrate concentrations differentiate humans with CDI from healthy controls. The SCFAs acetate, propionate, and butyrate were quantified in human stool samples from patients with symptomatic CDI and from healthy controls via GC-MS (see “Human subjects/patient enrollment” in Materials and Methods). Means are displayed as bars, standard deviations are displayed as error bars, and statistically significant differences in SCFA concentrations between each patient population were determined by Mann-Whitney test (*, *P* < 0.05).

After learning that butyrate concentrations differentiate mice with and without detectable C. difficile in their feces, we wanted to know if butyrate concentrations are similarly associated with CDI in humans. Though previous work showed that SCFA concentrations increase in stool from CDI patients after a fecal transplant (43), the differences in concentrations of SCFAs between humans with CDI and healthy controls was not previously determined. We quantified acetate, propionate, and butyrate in stool samples collected from patients who received care at Stanford Hospital in 2015. These stool samples were from patients with symptomatic CDI (diarrhea and positive for CDI [via Cepheid Xpert C. difficile]) and patients without CDI (negative for CDI [via Cepheid Xpert C. difficile]). In stool from the symptomatic C. difficile patients, we observed significantly lower concentrations of butyrate (but not acetate or propionate) than in stool from patients without CDI (Fig. 4), which demonstrates that our findings in mice (Fig. 3B) are generalizable to humans with CDI. Though acetate, propionate, and butyrate were previously shown to negatively impact the fitness of C. difficile and other bacterial pathogens (9, 44), our observations from mice and humans provide the rationale for focused and specific investigation of butyrate.

### Butyrate negatively impacts growth in diverse C. difficile isolates.

**FIG 5:**
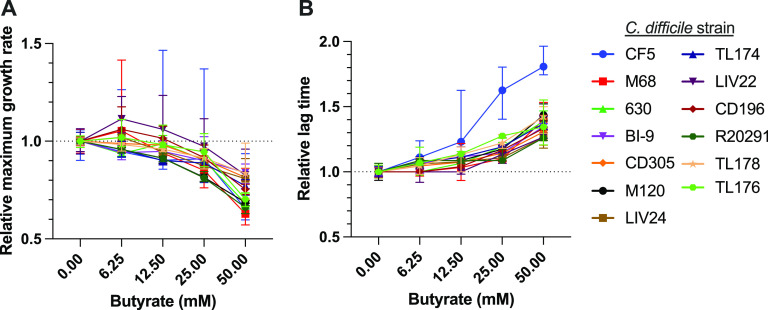
Butyrate negatively impacts growth in diverse C. difficile strains. Thirteen C. difficile strains (Table 1) were grown anaerobically in mRCM supplemented with either 0, 6.25, 12.5, 25, or 50 mM sodium butyrate or matched concentrations of sodium chloride (NaCl) for 24 h. Culture density (OD_600_) was monitored throughout this time course (*n* = 6 replicates per growth condition per strain). For all cultures, maximum growth rate (A) and lag time (B) were calculated. All strains tested had significantly longer lag times in the presence of 50 mM butyrate than in the presence of 0 mM butyrate. Similarly, all but 2 strains tested (CD196 and TL178) exhibited significantly reduced maximum growth rates in the presence of 50 mM butyrate compared to 0 mM butyrate. The median line is displayed, standard deviations are displayed as error bars, and statistically significant differences between relevant groups were determined by Mann-Whitney test, where a *P* value of <0.05 is considered statistically significant. Figure S2 in the supplemental material shows representative growth curves for all 13 strains under the growth conditions tested.

Our previous work showing that butyrate impacts C. difficile growth was restricted to the commonly studied C. difficile 630 strain (9) (Fig. 1 and 3). Though similar butyrate-dependent effects were observed in 4 unsequenced C. difficile isolates (20), we sought to further situate these findings in the context of a phylogenetically diverse sample of C. difficile strains. We grew 13 different C. difficile isolates with representatives from 10 ribotypes (including C. difficile 630 [Table 1]) in pure culture in the presence of 0, 6.25, 12.5, 25, and 50 mM sodium butyrate and in matched concentrations of sodium chloride. For all C. difficile strains tested, butyrate negatively impacts growth kinetics (see Fig. S2 in the supplemental material), with notable concentration-dependent differences in maximum growth rate (Fig. 5A) and lag time (Fig. 5B). All strains tested had significantly longer lag times in the presence of 50 mM butyrate than in the presence of 0 mM butyrate (Mann-Whitney test, *P* ≤ 0.05). Similarly, all but 2 strains tested (CD196 and TL178) exhibited significantly reduced maximum growth rates in the presence of 50 mM butyrate compared to 0 mM butyrate. The significance and magnitude of these effects were smaller for intermediate butyrate concentrations but were concentration dependent.

**TABLE 1:** Clostridioides difficile strains used in this study^*a*^

Strain	Ribotype	Place/date of isolation/source	Reference
630	12	Zurich/1982/human	54
BI-9	1	Gerding Collection	53
CD196	27	France/1985/human	56
CD305	23	London/2008/human	52
CF5	17	Belgium/1995/human	53
Liv022	106	Liverpool/2009/human	52
Liv024	1	Liverpool/2009/human	52
M120	78	UK/2007/human	53
M68	17	Dublin/2006/human	53
R20291	27	London/2006/human	56
TL174	15	Cambridge, UK/2009/human	52
TL176	14	Cambridge, UK/2009/human	52
TL178	2	Belfast/2009/human	52

a Related to Fig. 1, 2, 3, and 5.

Though bulk measurements of butyrate in human and mouse samples are lower than 50 mM (Fig. 2 to 4) (9, 43), concentrations of butyrate produced by microbiome members in the gut at relevant spatial scales (e.g., when C. difficile is in close proximity to butyrate-producing commensals) remain unclear but are likely higher than what is observed via bulk measurements. Regardless, the concentration-dependent effects we observe for all strains (Fig. 5) demonstrate that C. difficile growth is reliably impacted by butyrate and suggest that the molecular mechanisms underlying this response are conserved across diverse C. difficile strains.

## DISCUSSION

This work adds to the growing body of literature demonstrating that diet impacts animal models of CDI. Specifically, it refines previous observations about the impacts of MACs on C. difficile fitness in the gut by showing that diets which lead to elevated butyrate production by the microbiome reduce burdens of C. difficile during infection. Taken together, our work and the work of others show that inulin, maltodextrin, and xanthan gum are purified MACs that consistently suppress CDI while FOS, 2′-FL, 6′-SL, and LNnT are purified MACs that do not suppress CDI (Fig. 3) (9, 10). Unlike a standard rodent diet that is a complex mixture of MACs (9), we show that a different complex mixture of MACs (gum arabic) does not suppress C. difficile burdens in mice (Fig. 3). Importantly, our work exclusively used conventionally reared Swiss-Webster mice. Given the combined impacts that microbiome configurations and host genetics have on metabolites used and produced by a given community (45), it is possible that the MAC sources that cleared CDI in our model would not clear CDI in the context of a different microbiome, host, or antibiotic perturbation (7). As such, future work should consider the impacts of dietary MACs on CDI and how these effects are influenced by variables like the microbiome (e.g., using mice humanized with stool from different donors) (7), the relative impacts of different antibiotic pretreatment regimes (e.g., antibiotic cocktail followed by clindamycin injection, clindamycin only, streptomycin only, or cefoperazone only) (8, 16), and host genetics (e.g., using resources like collaborative cross or diversity outbred mice) in order to identify the generalizability of our findings prior to designing dietary strategies for impacting CDI in humans (46).

C. difficile burdens and SCFA levels are unlikely to be the only parameters impacted by MACs during infection in our model, which highlights additional directions for future work. For example, though we observed that FOS does not suppress CDI in mice (Fig. 1), it was previously shown that FOS increases survival time in hamsters infected with C. difficile (11), but the mechanism of this protection was not defined. Our previous work showed transient increases in gastrointestinal inflammation (via histopathology) 2 to 4 days after a shift from a MAC-deficient diet either to standard rodent chow or to an inulin-containing diet in infected mice, perhaps due to elevated C. difficile toxin production (9, 39). In addition, immune-mediated effects (e.g., via microbiome-produced butyrate that results from MAC consumption by the host) were previously shown to protect mice from colitis but to not impact C. difficile burdens in a mouse model (47). Importantly, that study used a different strain of C. difficile, a different antibiotic pretreatment, and a different strain of mice than what we used in this work (47). Finally, although we and others showed that butyrate is negatively associated with C. difficile burdens and colitis, work from others suggests that these effects might not be generalizable to all experimental models (e.g., butyrate was not associated with disease severity in a study that focused on the impacts of dietary fat on CDI in mice) (8).

Taken together, more work is needed to determine the relative impacts of various dietary inputs (see the introduction), microbial metabolites, and host inflammation under a range of experimental conditions to best inform paths to translation. These future efforts will likely contribute to the formulation of specific diet-based strategies to simultaneously bolster the host immune response and reduce the fitness of C. difficile, possibly through the manipulation of SCFA levels (which influence inflammation [48] and colonocyte metabolism [47, 49]) or by directly impacting the mucosal immune system (e.g., via HMOs which can influence inflammatory cell populations [50] and positively impact barrier function [51]).

Because butyrate levels differentiate mice and humans that have CDI from those that do not (Fig. 2C, Fig. 3B, and Fig. 4), continued focus on this SCFA in the context of CDI will yield important insights into the biology of C. difficile, the ecology of CDI, and the abovementioned future therapeutic approaches. We and others previously showed that butyrate negatively impacts growth in 5 distinct C. difficile strains (9, 20), and in the current study we extend these findings to 12 additional C. difficile strains (Fig. 5; Table 1), together demonstrating that these phenotypes are generalizable across a large sample of C. difficile clinical isolates. Future research on the molecular mechanisms and genetic circuitry underlying the responses of C. difficile to butyrate will be necessary to understand the relative contributions of this metabolite to C. difficile fitness and the host immune response and the degree to which these contributions impact various aspects of CDI in the context of the complex metabolic and microbial milieu of the gut microbiome. In addition, continued research on these and other diet-driven effects on CDI is likely to yield insights that will aid in the development of specific and targeted manipulation of CDI, through either dietary intervention, therapeutic application of specific microbes (e.g., probiotics), or delivery of specific metabolites.

## MATERIALS AND METHODS

### Bacterial strains and culture conditions.

Frozen stocks of C. difficile strains used in the study (Table 1) (52–54) were maintained as −80°C stocks in 25% glycerol under anaerobic conditions in septum-topped vials. C. difficile was routinely cultured on CDMN agar (Clostridioides difficile agar with moxalactam and norfloxacin), composed of C. difficile agar base (Oxoid) supplemented with 7% defibrinated horse blood (HemoStat Laboratories), 32 mg/L moxalactam (Santa Cruz Biotechnology), and 12 mg/L norfloxacin (Sigma-Aldrich) in an anaerobic chamber at 37°C (Coy).

After 16 to 24 h of growth, a single colony was picked into 5 mL of prereduced reinforced clostridial medium (RCM; Oxoid), modified reinforced clostridial medium (mRCM; 10 g/L beef extract, 3 g/L yeast extract, 10 g/L peptone, 5 g/L dextrose, 5 g/L sodium chloride, 3 g/L sodium acetate, 0.5 g/L cysteine hydrochloride), or PETC medium (ATCC medium 1754) without fructose (PETC-F), and grown anaerobically at 37°C for 16 to 24 h. Liquid cultures were used as inocula for growth curves and for experiments using murine model CDI, described below.

For *in vitro* growth curve experiments examining C. difficile fructan utilization, subcultures were prepared at a 1:200 dilution in prereduced PETC-F minimal medium supplemented with either 5 mg/mL inulin (Orafti HP; Beneo-Orafti) or 5 mg/mL FOS (Orafti P95; Beneo-Orafti) in sterile polystyrene 96-well tissue culture plates with low evaporation lids (Falcon). Cultures were grown anaerobically as described above in a BioTek PowerWave plate reader. At 15-min intervals, the plate was shaken on the “slow” setting for 1 min and the optical density at 600 nm (OD_600_) of the cultures was recorded using Gen5 software (version 1.11.5). After 24 h of growth, culture supernatants were collected, centrifuged (5 min at 2,500 × *g*), filtered (0.22-μm polyvinylidene difluoride [PVDF] filter), and stored at −20°C for high-performance anion-exchange chromatography, described below.

For *in vitro* growth curve experiments examining C. difficile growth in the presence of butyrate, subcultures were prepared at a 1:200 dilution in prereduced mRCM (RCM lacking starch and agar, which reduces clumping artifacts in OD_600_ readings) in sterile polystyrene 96-well tissue culture plates with low evaporation lids (Falcon). Cultures were grown anaerobically in a BioTek Epoch2 plate reader. At 30-minute intervals, the plate was shaken on the “slow” setting for 1 min and the OD_600_ of the cultures was recorded using Gen5 software (version 1.11.5).

### Murine model of C. difficile infection.

All animal studies were conducted in strict accordance with Stanford University Institutional Animal Care and Use Committee (IACUC) guidelines. Murine model CDI was performed on age- and sex-matched conventionally reared Swiss-Webster mice (Taconic) between 8 and 17 weeks of age.

To reduce colonization resistance to C. difficile, mice were given a single dose of clindamycin by oral gavage (1 mg/mouse; 200 μL of a 5-mg/mL solution) and were infected 24 h later with 200 μL of overnight culture grown in RCM (approximately 1.5 × 10^7^ CFU/mL).

Feces were collected from mice directly into microcentrifuge tubes and immediately placed on ice. To monitor C. difficile burdens in feces, 1 μL of each fecal sample was resuspended in phosphate-buffered saline (PBS) to a final volume of 200 μL, and 10-fold serial dilutions of fecal slurries (through 10^−3^-fold) were prepared in sterile polystyrene 96-well tissue culture plates (Falcon). For each sample, two 10-μL aliquots of each dilution (technical replicates) were spread onto CDMN agar supplemented with erythromycin (100 mg/L; Acros Organics). Erythromycin supplementation further reduces growth of bacteria from mouse feces and has no impact on C. difficile 630 colony counts (data not shown). After 16 to 24 h of anaerobic growth at 37°C, colonies were enumerated and technical replicates were averaged to determine C. difficile burdens in feces (limit of detection = 2 × 10^4^ CFU/mL feces). Immediately following euthanasia at 19 days postinfection, cecal contents were removed from mice, weighed, and flash frozen in liquid nitrogen. C. difficile was undetectable in all mice prior to inoculation with CDI.

### Mouse diets.

Mice were fed one of eight custom diets (Bio Serv) *ad libitum*: (i) a MAC-deficient control diet containing 68% (wt/vol) glucose, 18% (wt/vol) protein, and 7% (wt/vol) fat (MD; Bio-Serv) or diets containing 10% (wt/vol) amounts of one of the following ingredients as a sole source of MAC—(ii) inulin (Orafti HP; Beneo-Orafti, Mannheim, Germany), (iii) FOS (Orafti P95; Beneo-Orafti, Mannheim, Germany), (iv) gum arabic (Nutriloid gum arabic FT; TIC Gums, Belcamp, MD), (v) digestion-resistant maltodextrin (Fibersol-2; ADM/Matsutani LLC, Chicago, IL), (vi) lacto-*N*-neotetraose (LNnT; Kyowa Hakko, Tokyo, Japan), (vii) 2′-fucosyllactose (2′-FL; Inalco SpA, Milan, Italy), or (viii) 6′-sialyllactose (6′-SL; Inalco SpA, Milan, Italy). HMOs were enzymatically (LNnT) or chemically (2′-FL, 6′-SL) synthesized. For MAC-containing diets, MAC ingredients were swapped for an equal quantity of glucose.

### Human subjects/patient enrollment.

Human stool samples were collected from patients receiving care at Stanford Health Care between January 2015 and November 2015 and participating in an institutional review board (IRB)-exempt quality improvement project aimed at understanding the rates of C. difficile transmission in hematopoietic stem cell transplant patients. Samples either were from the patient’s first postadmission bowel movement or were collected at a frequency no more than once every 7 days postadmission. Samples were collected and immediately assayed for C. difficile TcdB using the Xpert C. difficile assay (Cepheid). Patients with unformed, C. difficile-positive stools were considered to have CDI. After this diagnostic procedure, residual deidentified samples (regardless of CDI status) were stored at 4°C for no more than 48 h and frozen at −80°C. Samples were subjected to targeted metabolomics, where the SCFAs acetate, propionate, and butyrate were quantified (see “SCFA quantification,” below).

### Quantification of FOS degradation products.

To quantify FOS degradation by C. difficile, spent and noninoculated PETC-F medium supplemented with 5 mg/mL FOS was filtered through 0.22-μm PVDF filters, dialyzed through centrifuge filters (10-kDa-molecular-weight cutoff [MWCO]; Millipore), and diluted with deionized water to bring the concentration of carbohydrate sources to a concentration of 1 μg/μL. Samples were subjected to high performance anion-exchange chromatography on a Dionex ICS-5000 system with an AS-AP autosampler and a pulsed amperometric detector, using a Dionex CarboPak PA1 column (4 by 250 mm analytical; Thermo Scientific) with a corresponding 4- by 50-mm guard column. The following solvent gradient was used (A = 100 mM NaOH, B = 100 mM NaOH, 1 M NaOAc): 0 to 60 min, 5% to 45% B; 60 to 70 min, 45% to 75% B. To prepare the reference chromatograms shown in Fig. S1 in the supplemental material, individual 5-mg/mL solutions of fructose, glucose, sucrose, kestose, nystose, and FOS were prepared in distilled water, filtered through 0.22-μm PVDF filters, and subjected to HPAEC-PAD as described above.

### SCFA quantification.

Two methods were used to quantify SCFAs in cecal contents from mice and in human stool: (i) a gas chromatography-mass spectrometry (GC-MS)-based method used in our previous work (9) and (ii) a liquid chromatography-mass spectrometry (LC-MS)-based method developed to overcome restrictions on access to core facility equipment during the early stages of the COVID-19 pandemic at Stanford University.

**(i) GC-MS-based SCFA quantification.** Cecal contents from mice or human stool (70 to 150 mg) were suspended in a final volume of 600 μL in ice-cold ultrapure water and blended with a pellet pestle (Kimble Chase) on ice. The slurry was centrifuged at 2,350 × *g* for 30 s at 4°C, and 250 μL of the supernatant was removed to a septum-topped glass vial and acidified with 20 μL high-performance liquid chromatography (HPLC)-grade 37% HCl (Sigma-Aldrich). Diethyl ether (500 μL) was added to the acidified cecal supernatant to extract SCFAs. Samples were then vortexed at 4°C for 20 min on “high” and then were centrifuged at 1,000 × *g* for 3 min. The organic phase was removed into a fresh septum-topped vial and placed on ice. Then, a second extraction was performed with diethyl ether as described above. The first and second extractions were combined for each sample, and 250 μL of this combined solution was added to a 300-μL glass insert in a fresh glass septum-topped vial, and the SCFAs were derivatized using 25 μL *N*-tert-butyldimethylsilyl-*N*-methyltrifluoroacetamide (MTBSTFA; Sigma-Aldrich) at 60°C for 30 min.

Analyses were carried out using an Agilent 7890/5975 single-quadrupole GC-MS. Using a 7683B autosampler, 1-μL split injections (1:100) were made onto a DB-5MSUI capillary column (30-m length, 0.25-mm inside diameter [i.d.], 0.25-μm film thickness; Agilent) using helium as the carrier gas (1 mL/minute, constant flow mode). Inlet temperature was 200°C, and transfer line temperature was 300°C. GC temperature was held at 60°C for 2 min, ramped at 40°C/min to 160°C, and then ramped at 80°/min to 320°C and held for 2 min; total run time was 8.5 min. The mass spectrometer used electron ionization (70 eV), and scan range was *m/z* 50 to 400, with a 3.75-minute solvent delay. Acetate, propionate, and butyrate standards (20 mM, 2 mM, 0.2 mM, 0.02 mM, and 0 mM) were acidified, extracted, and derivatized as described above, were included in each run, and were used to generate standard curves to enable SCFA quantification.

**(ii) LC-MS-based SCFA quantification.** The LC-MS-based SCFA quantification method was adapted from reference 55. Briefly, cecal contents from mice (50 to 150 mg) were weighed on an analytical balance and diluted in extraction buffer containing: 80% HPLC-grade water (Fisher), 20% HPLC-grade acetonitrile (ACN; Fisher), and labeled isotopes of each SCFA measured (2.5 μM d3-acetic acid [Sigma-Aldrich], 1 μM propionic-3,3,3-d3 acid [CDN Isotopes], and 0.5 μM butyric-4,4,4-d3 acid [CDN Isotopes]). The volume of extraction buffer in microliters was 4 times the mass of cecal contents in milligrams for each sample. Acid-washed beads (150 μM to 212 μM; Sigma-Aldrich G1145-10G) were added to the samples, and the samples were shaken at 30 Hz for 10 min to homogenize and extract the metabolites. The samples were then incubated at −20°C for 1 h and subsequently centrifuged at 4°C for 5 min at 12,000 relative centrifugal force (rcf). Forty microliters of the supernatant was transferred to a 96-well plate to which were added 20 μL of 200 mM 3-nitrophenylhydrazine hydrochloride (Sigma-Aldrich; dissolved in 50% ACN and 50% water) and 20 μL of 120 mM 1-ethyl-3-(3-dimethylaminopropyl)carbodiimide hydrochloride (Pierce; dissolved in 47% ACN, 47% water, and 6% HPLC-grade pyridine [Sigma-Aldrich]). The plate was then sealed and shaken in an incubator at 37°C for 30 min. After 30 min the plate was cooled to 4°C and 20 μL of the reaction volume was transferred to 980 μL of a 90:10 (vol/vol) water-ACN solution.

Analyses were carried out using an Agilent 6470 triple-quadrupole LC-MS. Using a G7167B multisampler, 10-μL injections were made onto an Acquity ultraperformance liquid chromatography (UPLC) ethylene-bridged hybrid (BEH) C_18_ column (100-mm length, 2.1-mm inner diameter, 130-Å pore size, 1.7-μm particle size; Waters) using water-formic acid (100:0.01, vol/vol; solvent A) and acetonitrile-formic acid (100:0.01, vol/vol; solvent B) as the mobile phase for gradient elution. The column flow rate was 0.35 mL/min; the column temperature was 40°C, and the autosampler was kept at 5°C. The binary solvent elution gradient was optimized at 15% B for 2 min and 15% to 55% B for 9 min and then held at 100% B for 1 min. The column was equilibrated for 3 min at 15% B between injections. The drying gas (N_2_) temperature was set to 300°C with a flow rate of 12 L/min. The sheath gas temperature was also set to 300°C with a flow rate of 12 L/min. The nebulizer gas was set to 25 lb/in^2^, and the capillary voltage was set to 4,200 V.

Quantification of analytes was done by standard isotope dilution protocols. In brief, serial dilutions of a 3-SCFA standard solution (10 mM, 1 mM, 0.1 mM, 0.01 mM, 0.001 mM, and 0 mM) were derivatized as described above and included in each run to verify that sample concentrations were within linear ranges. For samples within linear range, analyte concentration was calculated as the product of the paired internal standard concentration and the ratio of analyte peak area to internal standard peak area. A single product ion was used for each analyte; no secondary or qualifier ions were used. To ensure the highest signal-to-noise ratio, the following steps were taken. First, to ensure that the predicted singly derivatized species was the dominant precursor ion, full-mass Q1 scans were performed over the *m/z* range 100 to 300. Second, collision energies and fragmentor voltage were optimized using Agilent’s MassHunter Optimizer program with direct infusion of the derivatives from individual standard solutions containing 50 mM (each) fatty acid. Optimizer was set to search collision energies from −10 V to −120 V in 10-V increments and to select the two most intense product ions for optimization. Fragmentor voltage had minimal impact and was manually set to 75 V.

### Measurement of maximum growth rate and lag time for *in vitro* growth experiments.

Raw OD_600_ measurements of cultures grown in mRCM (see “Bacterial strains and culture conditions,” above) were exported from Gen5 and analyzed using the growth_curve_statistics.py script (see “Code availability,” below). Growth rates were determined for each culture by calculating the derivative of natural log-transformed OD_600_ measurements over time. Growth rate values at each time point were then smoothed using a moving average over 150-min intervals to minimize artifacts due to noise in OD measurement data, and these smooth growth rate values were used to determine the maximum growth rate for each culture. To mitigate any remaining issues with noise in growth rate values, all growth rate curves were also inspected manually. Specifically, in cases where the growth_curve_statistics.py script selected an artifactual maximum growth rate, the largest local maximum that did not correspond to noise was manually assigned as the maximum growth rate. Additionally, lag time was calculated as half the time to reach the maximum growth rate.

### Code availability.

Python script that was used to compute maximum growth rate and lag time from growth curve data is freely available at https://github.com/HryckowianLab/Pensinger_2022.

### Statistical analysis.

Statistical analysis was performed using GraphPad Prism 9.1.0. Details of specific analyses, including statistical tests used, are found in the applicable figure legends. Significance is indicated as follows: *, *P* < 0.05; **, *P* < 0.01; ***, *P* < 0.001; ****, *P* < 0.0001.
