# Supplementary material for: Butyrate Differentiates Permissiveness to Clostridioides difficile Infection and Influences Growth of Diverse C. difficile Isolates
Source: Infect Immun. 2023 Jan 24;91(2):e00570-22. doi: 10.1128/iai.00570-22 (PMC9933713; doi:10.1128/iai.00570-22)
Supplement: Supplemental file 1 — Fig. S1 and S2. Download iai.00570-22-s0001.pdf, PDF file, 0.8 MB [file iai.00570-22-s0001.pdf]

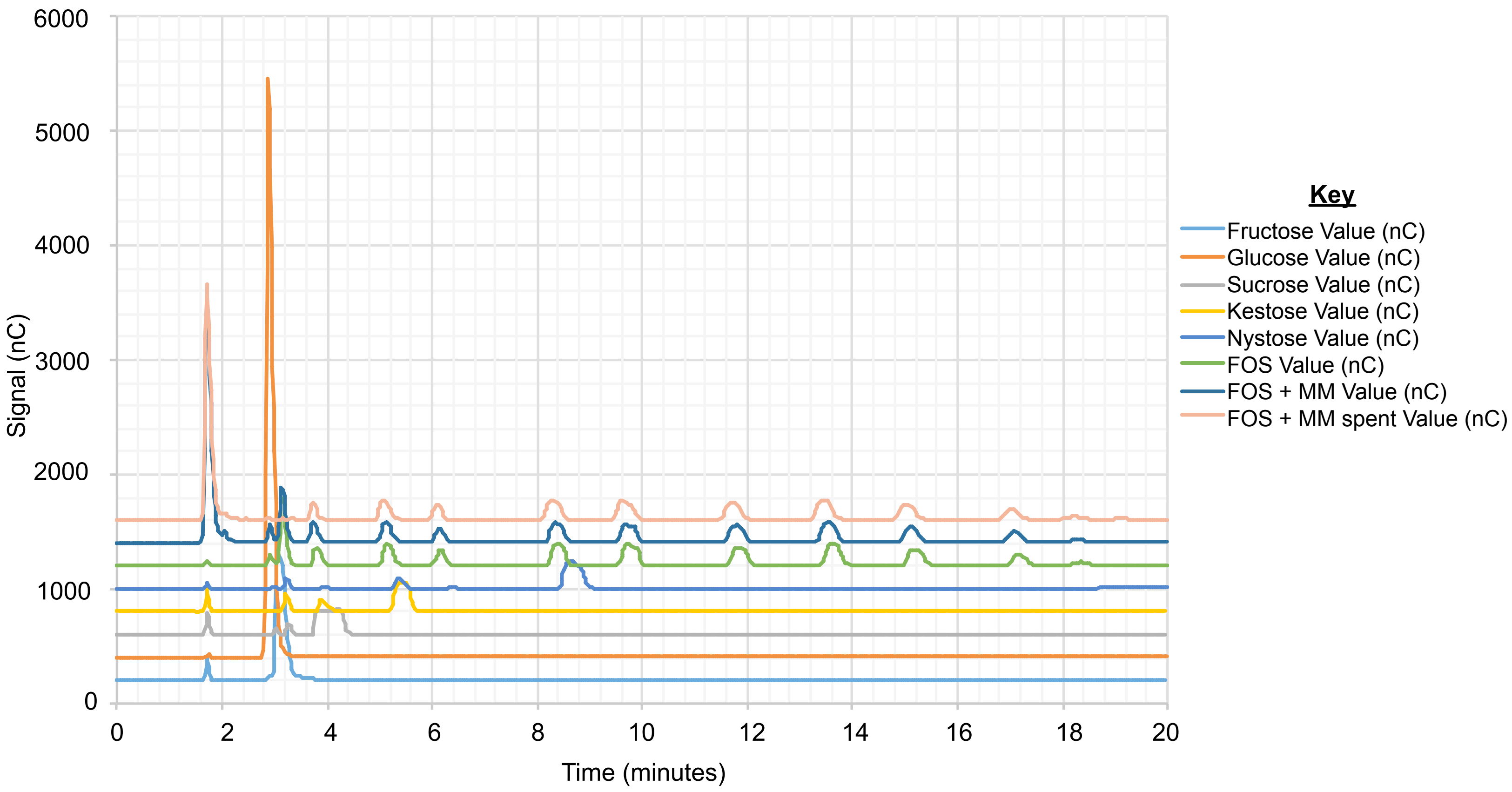

Supplemental Figure 1. HPAEC-PAD Chromatograms. Reference chromatograms for fructose (light blue), glucose (orange), sucrose (gray), kestose (yellow), nystose (blue), and FOS (green) are shown. Chromatograms for FOS-supplemented PETC-F minimal medium (FOS + MM; dark blue) and spent PETC-F minimal medium (FOS + MM spent; peach) are also shown and duplicated from **Figure 2B**. Related to **Figure 2.**

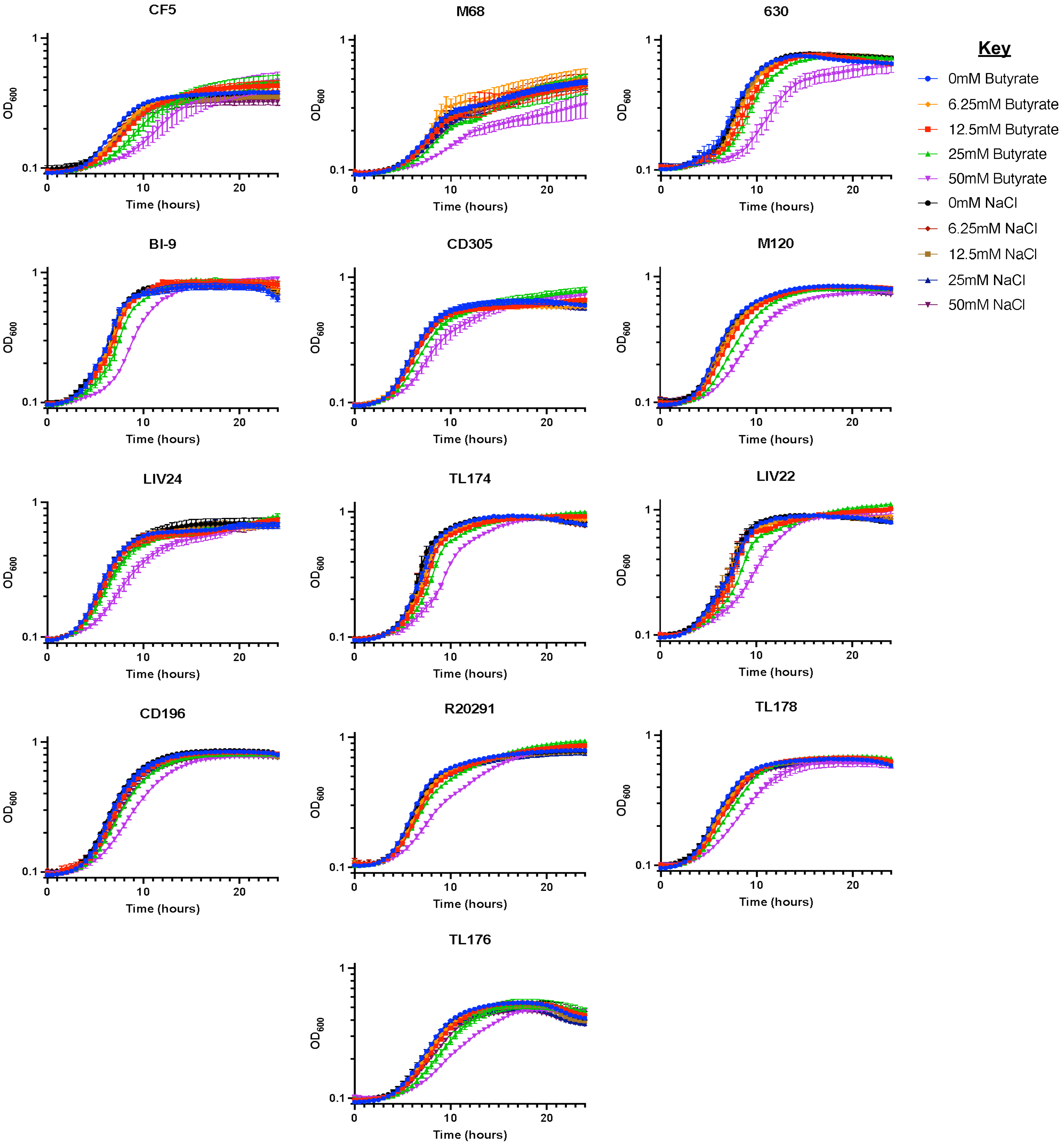

Supplemental Figure 2. Representative growth curves of thirteen *C. difficile* strains grown in the presence of sodium butyrate and sodium chloride. The thirteen *C. difficile* strains listed in **Table 1** were grown anaerobically in mRCM supplemented with either 0, 6.25, 12.5, 25, or 50mM sodium butyrate or identical concentrations of NaCl for 24 hours. Each plot shows three representative growth curves per strain per condition and represents raw culture density (OD_{600}) measurements for each strain tested. Symbols represent mean and standard deviation of replicates. Related to **Figure 5**.
